# Long-term tuina can inhibit the occurrence of gastroparesis by protecting gastrointestinal function in diabetic rats

**DOI:** 10.3389/fendo.2025.1536567

**Published:** 2025-06-25

**Authors:** Jian-zhen Jiang, Wan-qiu Li, Kun-lin Kuang, Yu-qing Jiang, Zhao-xuan He, Li-juan Zhang, Jing-ya Cao, Dan Wang, Xin-yue Zhang, Zi-lei Tian, Jun Zhu, De-zhong Peng

**Affiliations:** College of Acupuncture and Tuina, Chengdu University of Traditional Chinese Medicine, Chengdu, China

**Keywords:** long-term tuina, diabetes mellitus, diabetic gastroparesis, gastrointestinal function, Piezo2/5-HT pathway

## Abstract

**Background:**

Diabetic gastroparesis (DGP) is a common complication in the later stage of diabetes mellitus (DM). The aim of this study was to investigate the protective effect of long-term tuina on gastrointestinal (GI) function and the occurrence of DGP in diabetic rats.

**Methods:**

Twenty healthy male SD rats were randomly divided into four groups: NC, DM, DM + GT, and DM + TT. DM was induced with streptozotocin and a high-fat, high-sugar diet for 6 weeks. The DM + TT group received tuina therapy (20 min/session, 5 times/week) for 6 weeks. Weekly random blood glucose, gastric emptying rate, and small intestinal propulsion rate were measured. Hematoxylin and eosin (HE) staining assessed gastric antrum and ileum pathology. Ca^2+^-Mg^2+^-ATPase and CS activities, and neuronal nitric oxide synthase (nNOS), calmodulin (CaM), and myosin light chain kinase (MLCK) mRNA and protein expressions were evaluated by PCR and Western blot. 5-HT content was measured by ELISA. Piezo2 and 5-HT4R expressions were analyzed by immunofluorescence staining to observe tuina’s protective effect on GI function in DM rats.

**Results:**

Random blood glucose measurements showed normal levels in the NC group, while other groups remained above 16.7 mmol/L. The DM group exhibited reduced gastric emptying and small intestinal propulsion rates, along with gastric antrum and ileum damage. The DM + TT group showed significant improvements in gastric emptying and intestinal propulsion rates, and reduced tissue damage compared to the DM group. In the DM + TT group, mRNA and protein expressions of CaM and MLCK in antrum tissue, and nNOS, CaM, and MLCK in ileum tissue, were significantly increased. Activities of Ca^2+^-Mg^2+^-ATPase and CS enzymes in ileum tissue were also elevated, indicating enhanced GI function. Further analysis showed increased mRNA expressions of Piezo2 and 5-HT4R, and protein expressions of Piezo2, 5-HT, and 5-HT4R in the ileum tissues of the DM + TT group. Immunofluorescence intensity of Piezo2 and 5-HT in the ileum was also heightened. These results suggest that tuina’s protective effect on GI function is related to the expression of Piezo2 ion channels.

**Conclusions:**

Long-term tuina protects the GI function of DM rats and inhibits the occurrence of DGP, which might be related to the Piezo2/5-HT pathway.

## Introduction

Diabetic gastroparesis (DGP) is a common complication in the later stage of diabetes mellitus (DM). It refers to a disorder characterized by delayed gastric emptying and upper abdominal discomfort, which is caused by gastrointestinal (GI) motility dysfunction without mechanical obstruction of the GI tract. The main manifestations are early satiety, nausea, vomiting, abdominal distension, and other symptoms, which seriously affect the quality of life of patients (1–3). Also, these GI symptoms may originate not only from the stomach but also from the small intestine (1, 4).

The digestive functions of the GI tract, such as gastric emptying and small intestinal peristalsis, depend on the coordinated secretion and movement of fluid in response to mechanical stimulation within the lumen (5). Studies have shown that force can cause neuroendocrine cells to release 5-hydroxytryptamine (5-HT) (6–8). Mechanical stimulation of the GI mucosa results in 5-HT release (9–11), which stimulates fluid secretion (11, 12) and GI motility (13). Enterochromaffin (EC) cells are the largest group of intestinal epithelial enteroendocrine (EE) cells. EC cells are thought to be specialized mechanosensory cells that release 5-HT in response to epithelial forces, thereby regulating intestinal fluid secretion (5, 14). The mechanical sensitivity of the ion channel Piezo2 acts as touch and proprioception receptors, expressed in the EC cells of mice and humans (8, 15–18). Piezo2 is required for sensing intestinal contents and slowing the rate of food transport in the stomach, small intestine, and colon, and humans lacking Piezo2 exhibit impaired GI sensation and motility (19). Piezo2 mechanosensors are essential for the coupling force of 5-HT release and intestinal secretion (14).

Tuina therapy is a mechanical therapy with regular, rhythmic movement and more power than touch, which is applied to the surface skin or muscles and has been widely used in alternative and complementary medicine (20, 21). Tuina therapy can reduce gastric retention, reduce the gastric residual volume (22, 23), improve gastric antrum and feeding intolerance, and reduce the incidence of abdominal distension, vomiting, and diarrhea (24–27). It can also improve the overall symptoms and constipation symptoms of patients with functional dyspepsia (28–31).

However, we lack sufficient understanding of the molecular mechanisms of tuina therapy in alleviating GI dysfunction, especially the mechanism of long-term tuina therapy in protecting gastric emptying, and small intestinal propulsion function is still unclear. Piezo2 ion channel is considered to play a role in Gl diseases and its potential impact on tuina. We hypothesize that long-term tuina improves GI function in diabetic rats by modulating the Piezo2/5-HT signaling pathway. By using real-time quantitative PCR, Western blot (WB), and immunofluorescence technology, we demonstrate the important role of Piezo2 in helping the gut to sense the mechanical force stimulus of tuina and thus modulate GI function.

## Methods

### Experimental animals and grouping

A total of 20 healthy SPF male Sprague-Dawley rats, aged 8 weeks, weighing 180–220 g, were provided by Sichuan Research Institute of Biomedical Industry and Technology [production license number: SYXK (Sichuan) 2020-199]. The animals were raised in the Sichuan Institute of Biomedical Industry Technology. The ambient temperature was 20–25°C, lights on at 8:00–20:00, the relative humidity was 45%–60%, and the animals were free to drink and eat. All animal feeding and experimental procedures comply with the Guidelines for the Management and Use of Laboratory Animals. The trial protocol of this study was implemented with reference to ARRIVE guidelines (32). This project passed the ethical review of the Animal Ethics Committee of Sichuan Research Institute of Biomedical Industry Technology (No. 2021-07-15).

### DM and DGP rat models

After 1 week of adaptive feeding, the rats were randomly assigned to four groups using a random number table method: the normal control group (NC group), the diabetes mellitus group (DM group), the diabetes mellitus + gentle touch group (DM + GT group), and the diabetes mellitus + tuina treatment group (DM + TT group), with 5 rats in each group.

**Figure 1:**
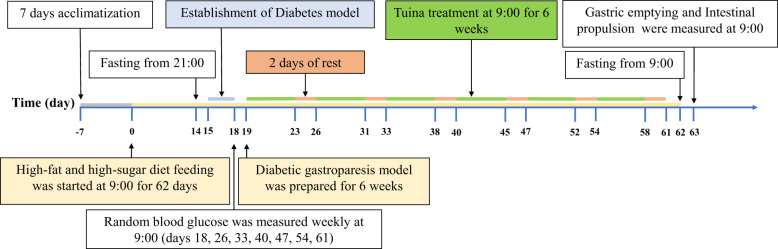
Experimental protocol. After 7 days of adaptive feeding, the rats in the DM group, DM + GT group, and DM + TT group were fed a high-fat and high-sugar diet for 62 days, and the rats in the NC group were fed a conventional diet. Fifteenth day STZ injection solution. Random blood glucose was measured every 7 days starting from day 18. After the DM model was successfully established, tuina treatment was started on the 19th day, 5 times a week for 6 weeks, for a total of 30 times. The 63rd day based measurement.

The modeling method of Srinivasan was used (33). The DM group, DM + GT group, and DM + TT group were fed a high-fat and high-sugar diet (60% fat, 20% protein, and 20% carbohydrate) and 5% sucrose water for 2 weeks. Two weeks later, streptozotocin (STZ) (35 mg/kg) was injected intraperitoneally after 12 h of fasting. The rats in the NC group were fed a normal diet and intraperitoneally injected with sodium citrate buffer (1 mL/kg). Blood samples were collected from the tail vein of the rats 72 hours after the injection to measure random blood glucose levels. The DM model was successfully established when the random blood glucose levels reached ≥16.7 mmol/L. The DM, DM + GT, and DM + TT groups were fed a high-fat and high-sugar diet and 5% sucrose water for 6 weeks. Random blood glucose was measured once a week during this period, and rats with random blood glucose < 16.7 mmol/L or dead rats were excluded (**Figure 1**).

Successful DGP modeling criteria (34): Random blood sugar is not less than 16.7 mmol/L. There were significant differences in hair color, behavior, mental state, and stool characteristics between the two groups. The semisolid gastric emptying rate and small intestinal propulsion rate in the DM group were lower than those in the NC group.

### Tuina treatment

After successful establishment of the DM model, rats in the DM + TT group received tuina therapy. The rats were immobilized using a custom-made rat tuina restraining device. Abdominal kneading: the operator’s right index finger and middle finger are naturally close together, and the rest of the palms are naturally flexed. The finger thread surface was attached to both sides of Shenque point (CV8), and clockwise rhythmic ring kneading was performed, with a frequency of 80 times/min, and the operation time was 10 min. Kneading Pishu (BL20): the operator’s right index finger and middle finger are naturally close together, and the rest of the palms are naturally flexed. The center of the finger thread surface was attached to the Pishu point (between the two ribs under the 12th thoracic vertebra, 6 mm away from the dorsal midline), the frequency of clockwise rhythmic ring kneading was 80 times/min, and the operation time was 10 min. The tuina therapy will be administered 5 times a week for a total of 30 sessions (**Figure 1**).

The DM + GT group, which serves as the sham tuina control, used a fine brush to perform gentle combing of localized hair areas. The fixing method and the position, frequency, and time of carding operation are consistent with the DM + TT group. The NC group and the DM group were fixed.

### Measurement of random blood glucose

All data collectors, testers, and analysts in this study were blinded to the grouping. The experiment was carried out at 9:00 a.m. on days 18, 26, 33, 40, 47, 54, and 61. Blood was collected from the tail of the rat while it was in a conscious state. Random blood glucose values were measured and recorded using a blood glucose meter and blood glucose test strips (ACCU-CHEK Performa, Roche Diagnostic Products Shanghai Co., Ltd., Shanghai, China).

### Measurement of gastric emptying rate and small bowel propulsive rate

After 6 weeks of tuina intervention, the rats were fasted for 24 h and fed 3 mL of semi-solid nutritional paste containing carbon powder (carboxymethylcellulose sodium 10 g, milk powder 16 g, starch 8 g, white sugar 8 g, activated carbon 2 g, dissolved in distilled water 250 mL mixed to make 300 mL, approximately 300 g of black semi-solid paste). Thirty minutes later, the whole stomach was weighed with intraperitoneal injection of 3% sodium pentobarbital at 5 mL·kg^−1^ under anesthesia. The stomach was cut along the greater curvature and rinsed thoroughly with 0.9% NaCl solution, the net weight of the stomach was weighed, and the gastric emptying rate was calculated. Gastric emptying rate = [1 − (total weight of stomach − net weight of stomach) ÷ weight of char powder semi-solid nutritional paste] ×100%. The total length of the small intestine (pylorus to ileocecal junction) and the propulsion length of the semi-solid nutritional paste containing charcoal powder were measured, and the propulsion rate of the small intestine was calculated. Intestinal propulsion rate = advancement distance of black paste measured from pylorus as starting point/total intestinal distance of small intestine ×100%.

### Observation by hematoxylin and eosin staining

Fresh gastric antral and ileal tissues were obtained and fixed in 4% paraformaldehyde solution (G1101, servicebio, Wuhan, China) for 24 h. The embedding machine (JB - P5, Wuhan Junjie Electronics Co., Ltd., Wuhan, China) was used for paraffin embedding. Sections (4 μm) were made on a microtome (HM325, Thermo Fisher Scientific, Waltham, MA, USA) and stained with hematoxylin and eosin (HE). The specimens were examined under a light microscope (400×), and the images were collected and analyzed.

### Enzyme activity was detected via the biochemical method

The ileal segment tissue was harvested and homogenized by adding ninefold homogenization medium, and the supernatant was used for quantification by BCA (BL521A, biosharp, Beijing, China). In accordance with the ATP enzyme kit (A016-1-1, Nanjing Jiancheng Bioengineering Research Institute Co., LTD., Nanjing, China) and citrate synthase (CS) kit (E-BC-K178-M, Elabscience Biotechnology Co., Ltd, Wuhan, China) operating instructions, biochemical detection analysis of Ca^2+^-Mg^2+^-atpase and CS enzyme activities is performed.

### Real-time quantitative PCR

Gastric antrum and ileal segment tissues were harvested for total RNA extraction using Trizol reagent. Synthetic cDNA was obtained using the AccuRT Genomic DNA Removal kit (G492, Applied Biological Materials Inc, Richmond, Canada) standard. Real-time quantitative PCR assays were performed using EvaGreen Express 2X qPCR MasterMix-No Dye (G891, Applied Biological Materials Inc., Richmond, Canada) on a fully automated medical PCR analysis system (Shanghai Hongshi Medical Technology Co., Ltd., Shanghai, China). Primer design methods and primer sequences are shown in **Table 1**.

**Table 1:** Primer sequence information.

Genes	Direction of direction	Primer sequence (5′-3′)	Length of product (bp)
Piezo2	Forward	ACGGATAGTGAAGAGGAGGAAGAAG	90
	Reverse	GGCTTGGTAGACGAACTGGAATG	
5-HT4R	Forward	GGCTGGAACAACATCGGCATAG	116
	Reverse	ACAGAGCAGGTGATGGCATAGG	
nNOS	Forward	AATGGTGGAGGTGCTGGAGGAG	112
	Reverse	GTCTGGAGAGGAGCTGATGGAGTAG	
CaM	Forward	ACGGTAATGGCACAATCGACTTC	106
	Reverse	TCAAACACACGGAACGCTTCTC	
MLCK	Forward	CTTCAAGATGGTGGTGGCTGTG	155
	Reverse	CTTCTGCTTGCTCCTTGTTCTCC	
β-Actin	Forward	TGTCACCAACTGGGACGATA	165
	Reverse	GGGGTGTTGAAGGTCTCAAA	

### Western blotting

Apply an adequate amount to the gastric antrum and ileum segment organization, using RIPA (BL504A biosharp, Beijing, China) pyrolysis liquid. Centrifuge for 10 minutes for the total protein solution. Protein concentrations were determined using the enhanced BCA protein kit (BL507A, biosharp, Beijing, China). Equal amounts of proteins were separated by sodium dodecyl sulfate polyacrylamide gel electrophoresis (SDS-PAGE) and transferred to PVDF membranes. The membranes were blocked with 5% skim milk for 1 h at room temperature. Primary antibodies Piezo2 (1:500, PA5-72976, Thermo Fisher Scientific, Waltham, MA, USA), 5-HT4R (1:1,000, DF3503, Affinity Biosciences Pty Ltd, Melbourne, Australia), neuronal nitric oxide synthase (nNOS) (1:1,000, AF6249, Affinity Biosciences Pty Ltd, Melbourne, Australia), calmodulin (CaM) (1:1,000, ab45689, abcam, Cambridge, UK), myosin light chain kinase (MLCK) (1:500, AF5314, Affinity Biosciences Pty Ltd, Melbourne, Australia), and β-Actin (1:5,000, AF7018, Affinity Biosciences Pty Ltd, Melbourne, Australia) were incubated overnight at 4°C. The specimens were incubated with the secondary Goat Anti-Rabbit IgG (H + L) HRP (1:10,000, GAR0072, Multisciences, Hangzhou, China) for 1 h at room temperature. In the chemidoc™ XRS imaging system (Bio-Rad Laboratories, Inc., Hercules, CA, USA) on the image.

### Enzyme-linked immunosorbent assay

Add a ninefold homogenate culture medium preparation to the ileal tissue. After centrifugation, the supernatant was extracted for quantification by BCA (BL521A, biosharp, Beijing, China). The content of 5-HT in ileum tissue was determined according to the instruction from the 5-HT ELISA kit (E-EL-0033c, Elabscience Biotechnology Co., Ltd, Wuhan, China). The OD values of each well were measured at a wavelength of 450 nm using a microplate reader.

### Immunofluorescence staining

The ileal segment tissue was fixed with 4% paraformaldehyde (G1101, servicebio, Wuhan, China) for 24 h. Embedding paraffin was acquired after ileal cross-sectional slices of the organization. Antigen repair was performed using citrate antigen repair solution (G1219, servicebio, Wuhan, China). The cells were incubated with 5% goat serum for 30 min. Primary antibodies Piezo2 (1:50, PA5-72976, Thermo Fisher Scientific, Waltham, MA, USA) and 5-HT (1:200, sc-58031, Santacruz Biotech, Texas, USA) were added and incubated overnight at 4°C. They were washed three times in phosphate-buffered saline (PBS) (G4202, biosharp, Beijing, China). The cells were incubated with secondary antibodies Cy3-conjugated Goat Anti-Rabbit IgG H&L (1:200, GB21303, servicebio, Wuhan, China) and FITC-conjugated Goat Anti-Rabbit IgG H&L (1:200, GB22303, servicebio, Wuhan, China) for 1 h at room temperature. Nuclei were counterstained with 4′,6-diamino-2-phenylindole (G1012, servicebio, Wuhan, China). Images were observed and collected under an ICX41 fluorescence microscope (Ningbo Sunny Instrument Co., Ltd., Ningbo, China) (400×).

### Statistical analysis

IBM SPSS 26.0 software (IBM Statistics, Armonk, NY, USA) was used for statistical analysis. GraphPad Prism 10 Software (GraphPad Software, Boston, MA, USA) was used for plotting. Measurement data were expressed as mean ± SEM. One-way analysis of variance (ANOVA) was used to test the differences between groups. If the data were normally distributed, followed by the Bonferroni post-hoc test was used for pairwise comparisons. Otherwise, Kruskal–Wallis test was performed, followed by the Tukey or Dunn test for pairwise comparisons. Repeated measurement data were analyzed by repeated-measures ANOVA. A *p*-value of less than 0.05 is considered statistically significant.

## Results

### Effect of long-term tuina on random blood glucose in DM rats

**Figure 2:**
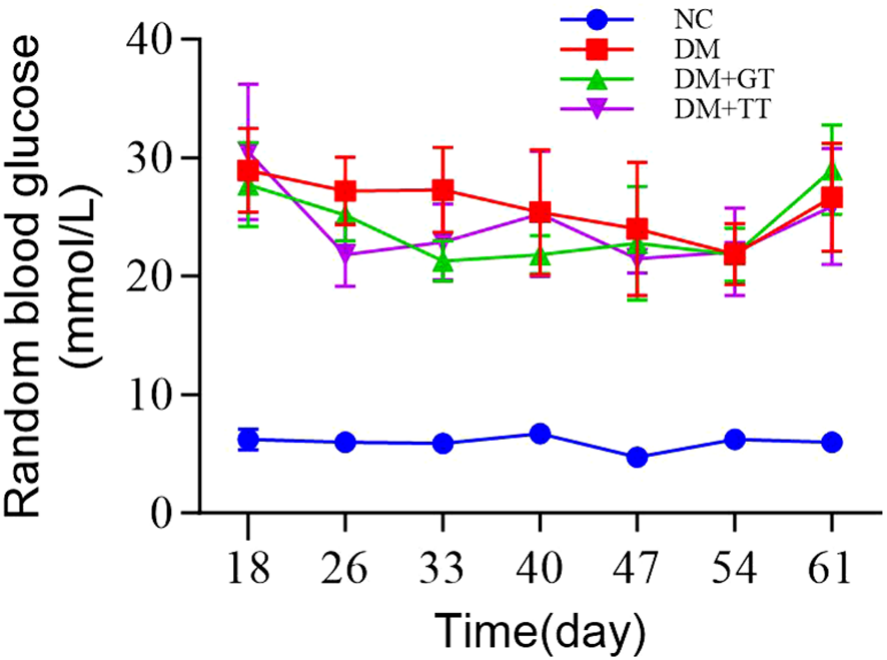
Groups of rats at different time points of random blood glucose. Data are expressed as mean ± standard deviation, *n* = 5. NC, normal control group; DM, diabetes mellitus group; DM + GT, diabetes mellitus + gentle touch group; DM + TT, diabetes mellitus + tuina treatment group.

During the long-term tuina treatment, random blood glucose detection was carried out once a week. The results showed that the rats in the DM group, DM + GT group, and DM + TT group were fed a high-fat and high-sugar diet for 6 weeks after the establishment of the DM model. The random blood glucose value of rats was maintained above 16.7 mmol/L, which met the basic conditions for the preparation of DGP GI dysfunction. There was no significant decrease in the DM + TT group compared with the DM group, which may be related to the continuous feeding of a high-fat and high-sugar diet (**Figure 2**).

### Long-term tuina protects the gastric emptying rate and small intestinal propulsion rate in DM rats

**Figure 3:**
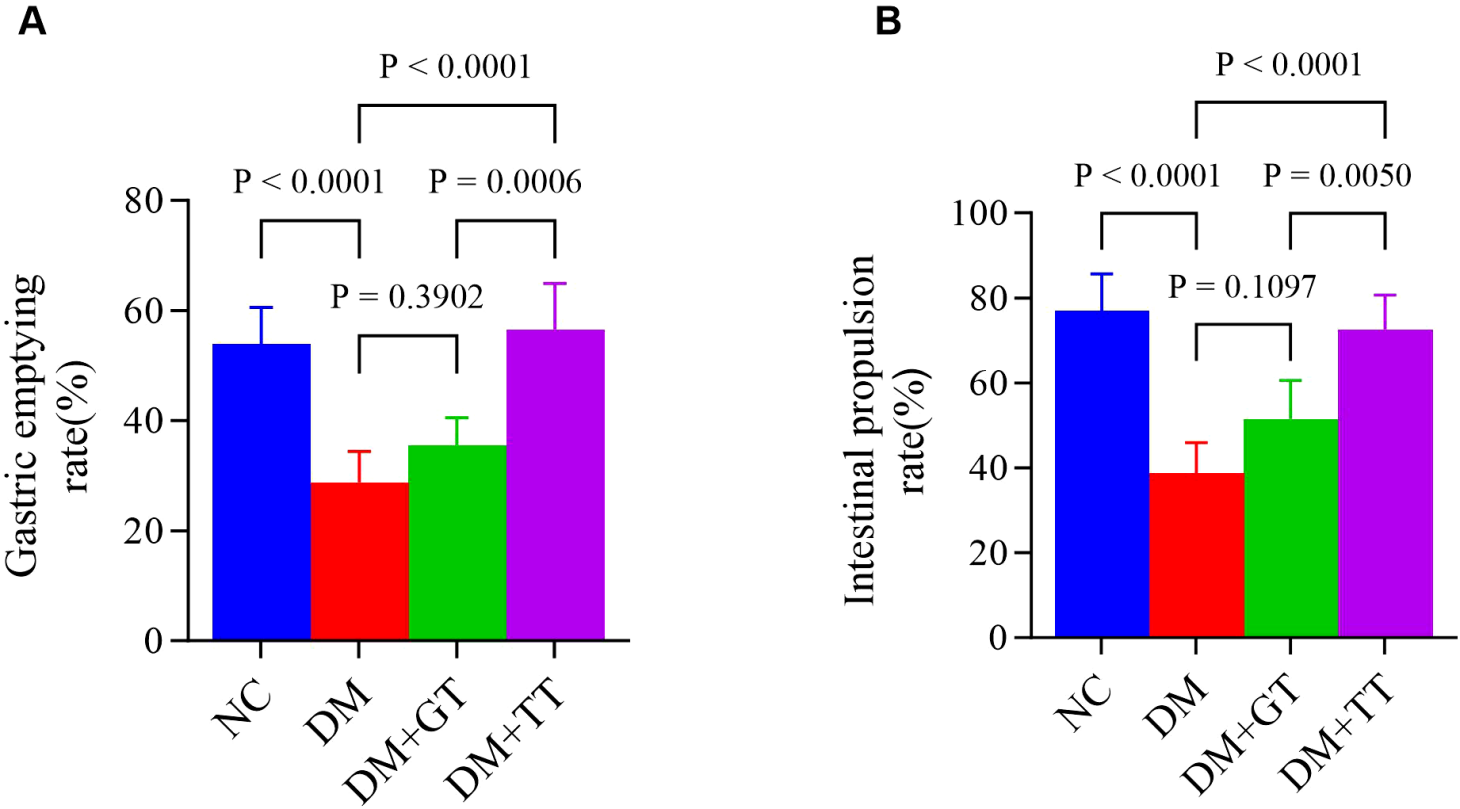
Comparison of gastric emptying rate and small intestinal propulsive rate in each group. **(A)** Gastric emptying rate. **(B)** Small intestinal propulsive rate. Data are expressed as mean ± standard deviation, *n* = 5. NC, normal control group; DM, Diabetes mellitus group; DM + GT, Diabetes mellitus + gentle touch group; DM + TT, Diabetes mellitus + tuina treatment group.

In order to observe the protective effects of long-term tuina on GI function in DM rats. After completing 30 tuina treatments, we measured the gastric emptying rate and the small bowel propulsion rate. Compared with the NC group, the gastric emptying rate (**Figure 3A**) and the small intestinal propulsion rate (**Figure 3B**) in the DM group were significantly decreased (*p* < 0.01). Compared with the DM group, the levels in the DM + TT group were significantly increased (*p* < 0.01). The DM + TT group was superior to the DM + GT group (*p* < 0.01). The results showed that long-term tuina could significantly protect gastric emptying and small intestinal propulsion in DM rats.

### Long-term tuina protects the morphology of GI tissue in DM rats

**Figure 4:**
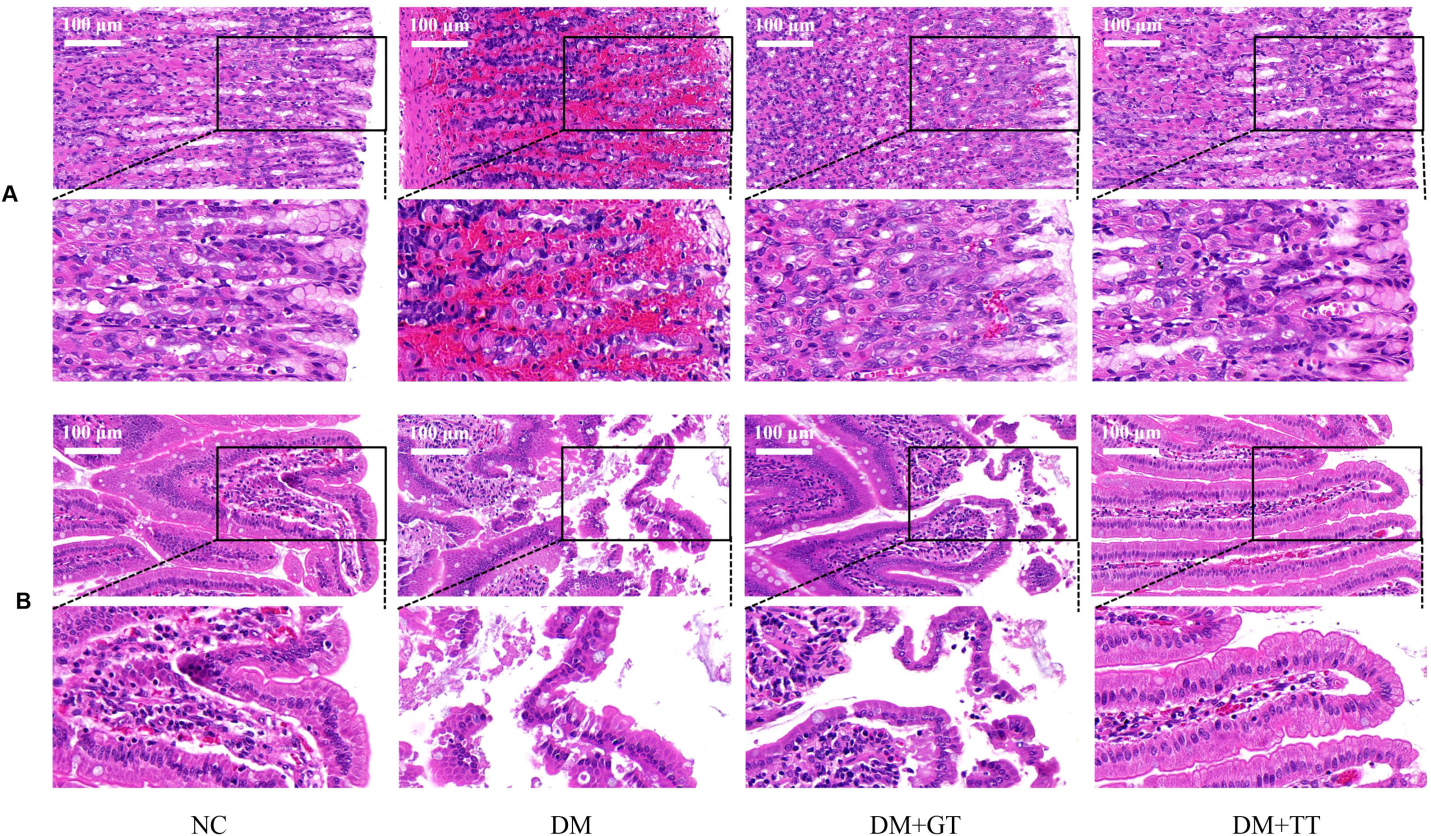
HE staining results of gastric antrum and ileum of rats in each group (400×). **(A)** Gastric antrum tissue. **(B)** Ileum tissue. NC, Normal control group; DM, Diabetes mellitus group; DM + GT, Diabetes mellitus + gentle touch group; DM + TT, Diabetes mellitus + tuina treatment group.

To observe the effect of long-term tuina on the morphology of GI tissue in DM rats, we performed HE staining of tissue from the antrum and ileum (**Figure 4**). There were no obvious pathological changes in the gastric antrum and ileum in the NC group and the DM + TT group. In the DM group, gastric mucosal hemorrhage, necrosis and exfoliation of mucosal epithelial cells, cell vacuolization, and inflammatory cell infiltration were observed. The structure of intestinal mucosa was destroyed, the apical structure of villi was dissolved, and the epithelial cells were necrotic and exfoliated. Vacuolization of gastric epithelial cells and edema of submucosa were observed in the DM + GT group. Local exfoliation of villous epithelial cells and histolysis were observed in the intestinal mucosa.

### Long-term tuina upregulates the expression of CaM and MLCK in the gastric antrum tissue of DM rats

**Figure 5:**
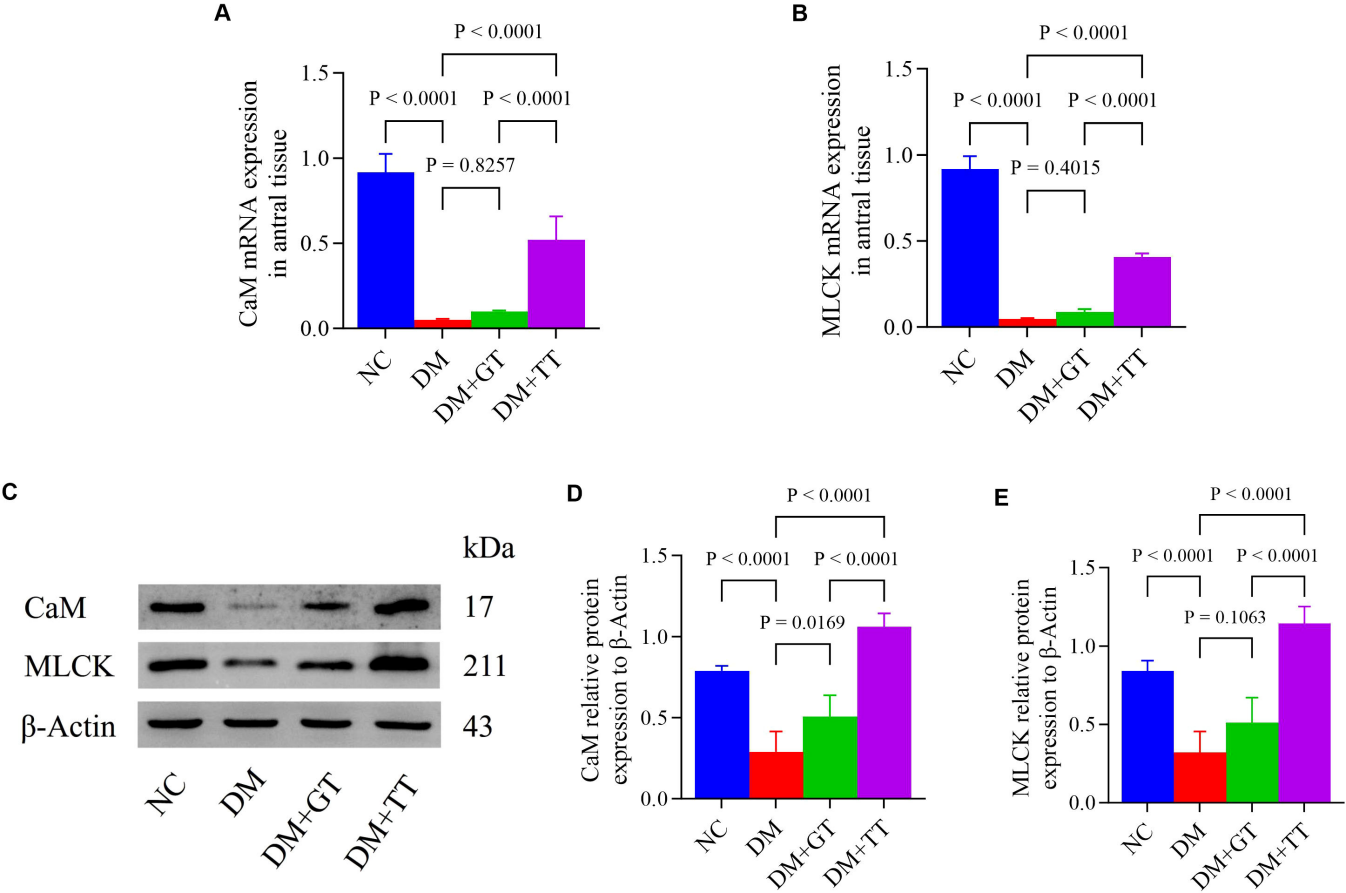
mRNA and protein expression of CaM and MLCK in gastric antrum tissues of rats in each group. **(A, B)** Gastric antrum tissue CaM and MLCK mRNA expression. **(C–E)** Expression of CaM and MLCK protein in gastric antrum tissue. Data are expressed as mean ± standard deviation, *n* = 5. NC, Normal control group; DM, Diabetes mellitus group; DM + GT, Diabetes mellitus + gentle touch group; DM + TT, Diabetes mellitus + tuina treatment group. CaM, Calmodulin; MLCK, Myosin light chain kinase.

CaM and MLCK molecules play an important role in regulating the contraction behavior of GI smooth muscle through the CaM/MLCK pathway (35). In order to further verify the regulatory effect of long-term tuina on gastric motility, antral tissue was assayed for CaM and MLCK mRNA and protein by RT-qPCR (**Figures 5A, B**) and WB (**Figures 5C–E**). Compared with the NC group, the mRNA and protein expressions of CaM and MLCK in the gastric antrum tissue of the DM group were significantly decreased (*p* < 0.01). Compared with the DM group, the expression of CaM protein in the DM + GT group was significantly increased (*p* < 0.05), and the expression of CaM and MLCK mRNA and protein in the DM + TT group was significantly increased (*p* < 0.01). Compared with the DM + GT group, the mRNA and protein expressions of CaM and MLCK in the DM + TT group were increased (*p* < 0.01).

### Long-term tuina upregulates the expression of nNOS, CaM, and MLCK in ileum of DM rats

**Figure 6:**
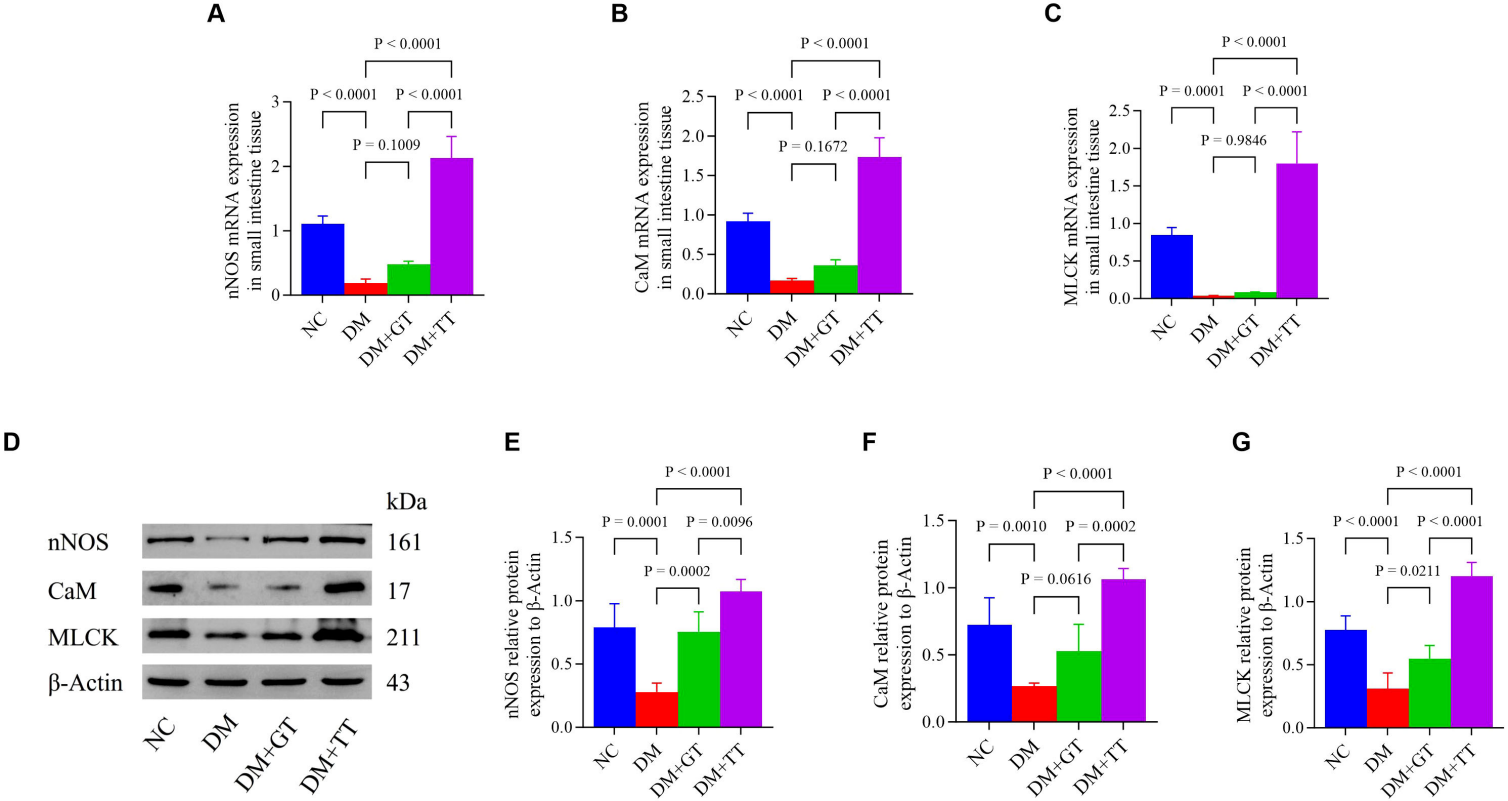
Expression of nNOS, CaM, and MLCK mRNA and protein in the ileum tissue of rats in each group. **(A–C)** Expression of nNOS, CaM, and MLCK mRNA in ileum tissue. **(D–G)** Protein expression of nNOS, CaM, and MLCK in ileum tissue. Data are expressed as mean ± standard deviation, *n* = 5. NC, Normal control group; DM, Diabetes mellitus group; DM + GT, Diabetes mellitus + gentle touch group; DM + TT, Diabetes mellitus + tuina treatment group. nNOS, Neuronal nitric oxide synthase; CaM, Calmodulin; MLCK, Myosin light chain kinase.

nNOS is involved in the maintenance of normal intestinal peristalsis. To verify the regulatory effect of long-term tuina on small intestinal motility, we examined the expression of nNOS, CaM, and MLCK molecules (mRNA: **Figures 6A–C**) (protein: **Figures 6D–G**) in ileal tissues. Compared with the NC group, the mRNA and protein expressions of nNOS, CaM, and MLCK in the DM group were significantly decreased (*p* < 0.01). Compared with the DM group, the protein expression of nNOS and MLCK in the DM + GT group was significantly increased (*p* < 0.01, *p* < 0.05). The mRNA and protein expressions of nNOS, CaM, and MLCK in the DM + TT group were significantly increased (*p* < 0.01). Compared with the DM + GT group, the mRNA and protein expressions of nNOS, CaM, and MLCK in the DM + TT group were significantly increased (*p* < 0.01).

### Long-term tuina upregulates the activities of Ca^2+^-Mg^2+^-ATPase and CS enzyme in the ileum of DM rats

**Figure 7:**
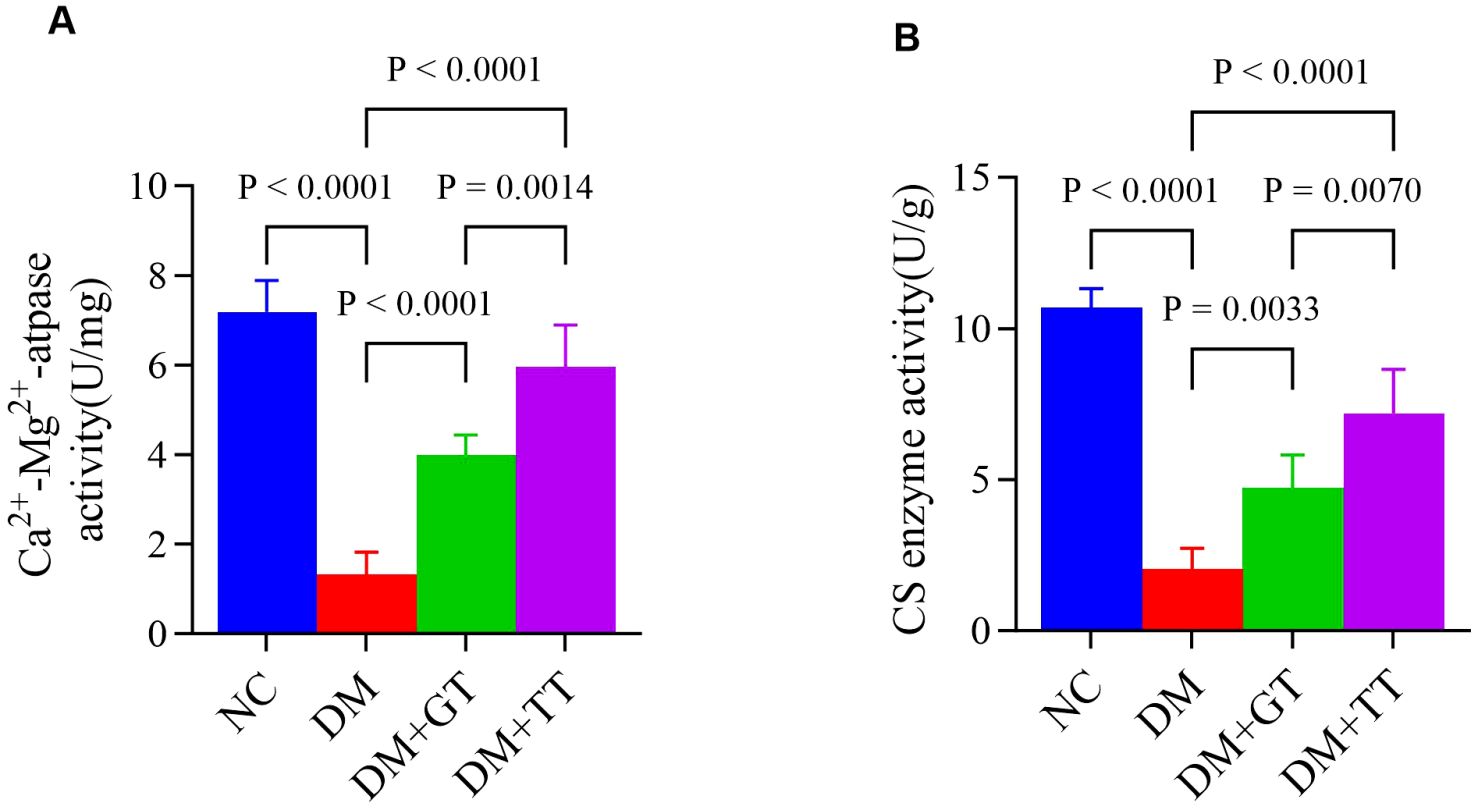
Comparison of ileum tissue enzyme activity between rat groups. **(A)** Activities of Ca^2+^-Mg^2+^-ATPase. **(B)** Activities of CS enzyme. Data are expressed as mean ± standard deviation, *n* = 5. NC, Normal control group; DM, Diabetes mellitus group; DM + GT, Diabetes mellitus + gentle touch group; DM + TT, Diabetes mellitus + tuina treatment group.

Ca^2+^-Mg^2+^-ATPase and CS enzyme activities are involved in energy metabolism in the regulation of GI motility. In order to further verify the regulatory effect of long-term tuina on small intestinal motility, we examined the Ca^2+^-Mg^2+^-ATPase (**Figure 7A**) and CS enzyme (**Figure 7B**) activities in ileal tissues. Compared with the NC group, the activities of Ca^2+^-Mg^2+^-ATPase and CS enzyme in the ileum tissue of the DM group were significantly decreased (*p* < 0.01). Compared with the DM group, the activities of Ca^2+^-Mg^2+^-ATPase and CS enzyme in the DM + GT group and DM + TT group were significantly increased (*p* < 0.01). Compared with the DM + GT group, the activities of Ca^2+^-Mg^2+^-ATPase and CS enzyme in the DM + TT group were higher (*p* < 0.01).

### Long-term tuina upregulates the expression of Piezo2 and 5-HT4R mRNA in the ileum tissue of DM rats

**Figure 8:**
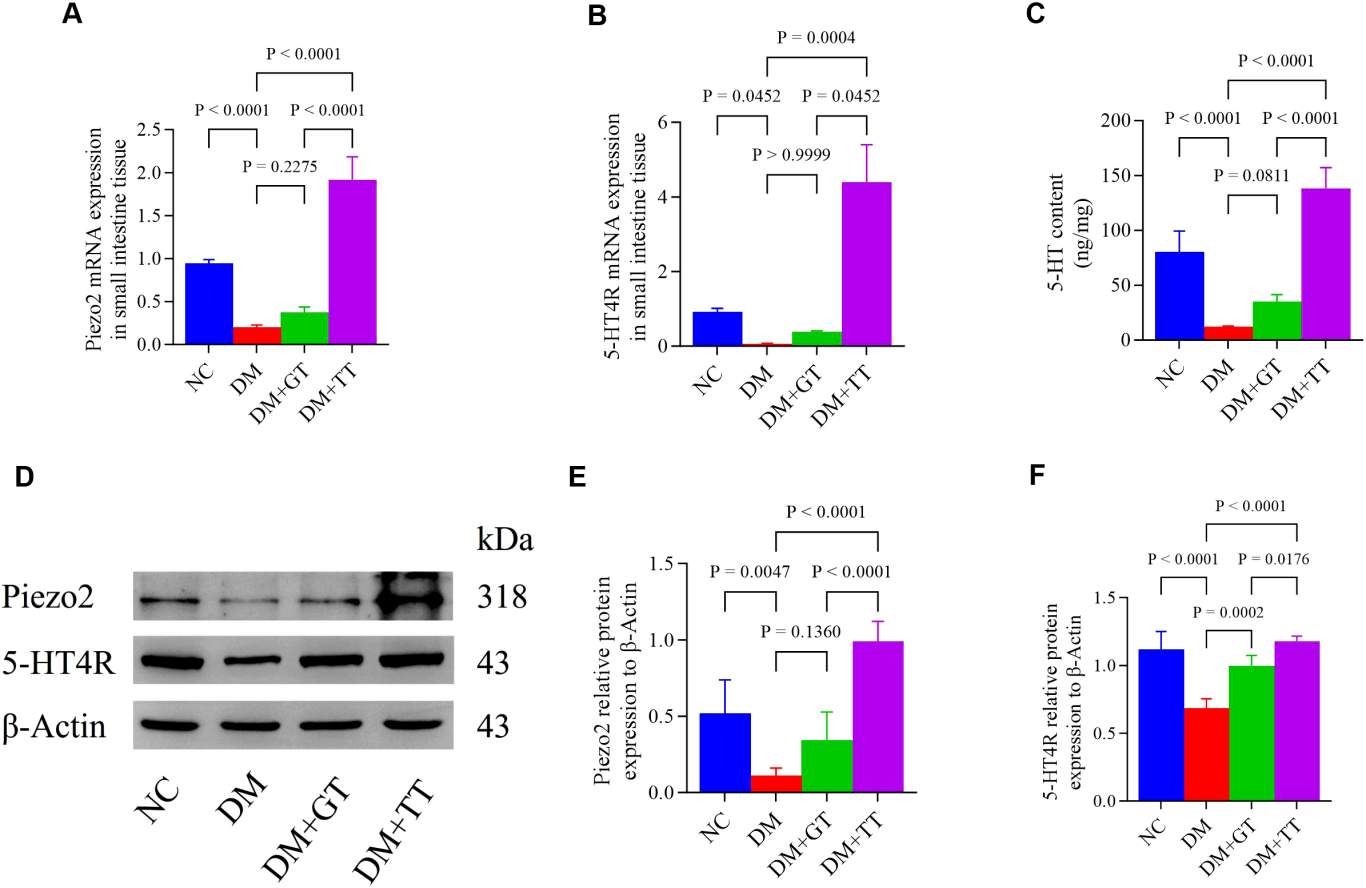
Ileum tissue Piezo2, 5-HT, and 5-HT4R protein expression in rat groups. **(A, B)** Expression of Piezo2 and 5-HT4R mRNA in ileum tissue. **(C)** Expression of 5-HT protein was detected by ELISA. **(D–F)** Western blotting was used to detect the expression of Piezo2 and 5-HT4R protein. Data are expressed as mean ± standard deviation, *n* = 5. NC, Normal control group; DM, Diabetes mellitus group; DM + GT, Diabetes mellitus + gentle touch group; DM + TT, Diabetes mellitus + tuina treatment group. Piezo2, The mechanically sensitive ion channel Piezo2; 5-HT, 5-hydroxytryptamine; 5-HT4R, 5-hydroxytryptamine 4 receptor.

To verify whether the protection of GI function by long-term tuina is related to the expression of Piezo2 and 5-hydroxytryptamine 4 receptor (5-HT4R) molecules, we examined Piezo2 and 5-HT4R mRNA expression in ileum tissue (**Figures 8A,B**). Compared with the NC group, the expression of Piezo2 and 5-HT4R mRNA in the DM group was significantly decreased (*p* < 0.01, *p* < 0.05). Compared with the DM group, the DM + TT group’s Piezo2, 5-HT4R mRNA expression was significantly increased (*p* < 0.01). Compared with the DM + GT group, the mRNA expression of Piezo2 and 5-HT4R in the DM + TT group was higher (*p* < 0.01, *p* < 0.05).

### Long-term tuina regulates the expression of Piezo2, 5-HT, and 5-HT4R protein in the ileum tissue of DM rats

To further verify whether long-term tuina protection of GI function is related to the expression of Piezo2, 5-HT, and 5-HT4R, which are important molecules regulating Piezo2/5-HT signaling pathway, we used WB, ELISA, and immunofluorescence staining techniques for detection. The results showed that compared with the NC group, the protein expression of Piezo2, 5-HT, and 5-HT4R in the ileum tissue of the DM group was significantly decreased (*p* < 0.01) (**Figures 8C–F**). Compared with the DM group, the expression of 5-HT4R protein in the DM + GT group was significantly increased (*p* < 0.01), and the expression of Piezo2, 5-HT, and 5-HT4R protein in the DM + TT group was significantly increased (*p* < 0.01). Compared with the DM + GT group, the protein expressions of Piezo2, 5-HT, and 5-HT4R in the DM + TT group were higher than those in the DM + GT group (*p* < 0.01, *p* < 0.05).

**Figure 9:**
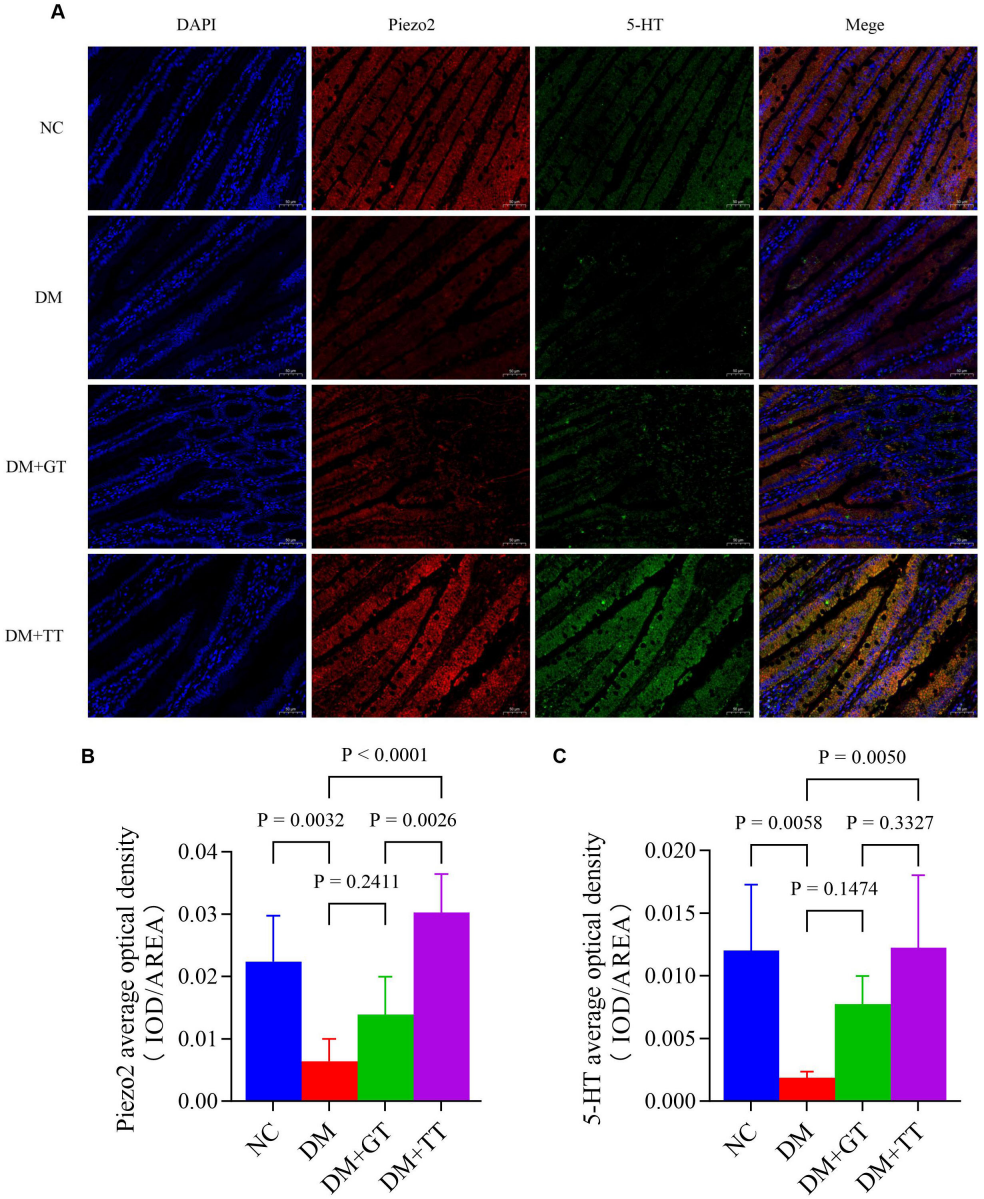
Immunofluorescence staining of Piezo2 and 5-HT in ileum tissue of rats in each group (400×). **(A)** Immunofluorescence staining. **(B)** Average optical density of Piezo2. **(C)** Average optical density of 5-HT. Data are expressed as mean ± standard deviation, *n* = 5. NC, Normal control group; DM, Diabetes mellitus group; DM + GT, Diabetes mellitus + gentle touch group; DM + TT, Diabetes mellitus + tuina treatment group. Piezo2, The mechanically sensitive ion channel Piezo2; 5-HT, 5-hydroxytryptamine.

Immunofluorescence staining results showed that compared with the NC group, the DM group’s ileum tissue, Piezo2, and 5-HT positive fluorescent expression reduced significantly (*p* < 0.01) (**Figures 9A, B**). Merge images showed that Piezo2 and 5-HT co-expression fluorescence was significantly reduced (**Figure 9**). Compared with the DM group, the positive fluorescence expression of Piezo2 and 5-HT in the DM + TT group was significantly increased (*p* < 0.01). Merge images showed that the co-expression fluorescence of Piezo2 and 5-HT was significantly increased. Compared with the DM + GT group, the positive fluorescence expression of Piezo2 in the DM + TT group was higher than that in the DM + GT group (*p* < 0.01).

## Discussion

In this study, it was confirmed that long-term abdominal and back tuina could significantly protect the gastric emptying function of DM rats, inhibit the deterioration of gastric antrum morphology, regulate the expression of CaM and MLCK in gastric antrum tissue, and slow down the progression of DGP. Meanwhile, we examined the small intestine propulsion rate; histological characteristics; the activities of Ca^2+^-Mg^2+^-ATPase and CS enzyme in the ileum; the mRNA and protein expression of nNOS, CaM, MLCK, Piezo2, and 5-HT4R; the protein expression of 5-HT in the ileum; and the co-expression of Piezo2 and 5-HT by immunofluorescence. The results showed that long-term tuina could not only significantly protect the gastric emptying function, but also protect the intestinal propulsion function, increase the activities of Ca^2+^-Mg^2+^-ATPase and CS in the ileum, and regulate the expression of nNOS, CaM, MLCK, Piezo2, 5-HT, and 5-HT4R.

The gentle touch intervention used as a sham tuina control is designed to mimic the effects of a light fingertip touch on the skin. It can regulate the expression of CaM protein in gastric antrum tissue, as well as the expression of MLCK and 5-HT4R proteins and the activity of Ca²^+^-Mg²^+^-ATPase and CS enzymes in ileal tissue. However, the gentle touch intervention does not significantly protect gastric emptying and small intestinal propulsion function.

The clinical manifestations of DGP and delayed gastric emptying may be caused by physiological changes, including dyskinesia, small bowel motility disorders, diminished pyloric relaxation, hypomotility of the antrum, and impaired regulation of the fundus/corpus (36, 37). A large number of studies confirm that the CaM/MLCK pathway on the GI smooth muscle contraction behavior plays an important regulatory role (35). When GI smooth muscle cells are stimulated by external electrical signals, extracellular Ca^2+^ enters the cell and binds to CaM to form a complex that activates CaM. The complex, in turn, activates MLCK, a key protein in calcium-dependent channels. This results in an increase in Mg^2+^-ATPase activity of the myosin head, hydrolyzes ATP, and provides energy for the relative sliding of myosin to actin, causing GI smooth muscle contraction (38–40). Our findings suggest that tuina therapy modulates CaM and MLCK expression in the gastric antrum tissue, which may contribute to improved GI smooth muscle contraction. However, whether these molecular changes directly mediate the observed functional improvements requires further investigation through measurements of intracellular Ca^2+^ dynamics and contractile force. nNOS plays a crucial role in intestinal peristalsis, and Akt phosphorylation of nNOS regulates ileogastric peristalsis in mice and induces cGMP-dependent GI smooth muscle relaxation, thereby promoting digestion (41–44). Hyperglycemia can reduce the number of intestinal neurons and the expression of nNOS mRNA and protein in the GI tract, which is the main factor causing abnormal intestinal motility. Restoring the expression of nNOS mRNA and protein in the GI tract can help reverse the delayed gastric emptying (45, 46).

Ca^2+^-Mg^2+^-ATPase and CS enzyme play a crucial role in maintaining the normal metabolic function of the body. Ca^2+^-ATPase is responsible for transporting Ca^2+^ from the cytoplasm to the mitochondria to ensure the maintenance of Ca^2+^ homeostasis, and the decrease of ATPase activity will destroy the intracellular energy metabolism (47–51). We observed a significant increase in the activity of these enzymes in the ileal tissue, which reflects an enhancement in the motility function of the small intestine.

DGP is more closely related to the 5-HT receptor. 5-HT distributed throughout the GI tract of the 5-HT receptor regulates GI function (52). 5-HT4 agonists were found to successfully treat various functional GI motility problems, such as constipation, constipation-predominant irritable bowel syndrome, functional dyspepsia, and DGP (53). A large number of studies have confirmed that the Piezo2/5-HT pathway is involved in the regulation of GI motility. EC cells are rare excitable, serotonergic neuroendocrine cells of intestinal epithelial cells that detect and transmit stimulus signals to nearby mucosal nerve terminals (5, 54–56). Meanwhile, in mouse small and large intestinal epithelial cells, Piezo2 is expressed in a subset of EC cells and localized on the membrane adjacent to 5-HT vesicles, where Piezo2 is essential for the coupling force of EC cell 5-HT release and enteroendocrine function (14).

Tuina therapy is a mechanical therapy of force stimulation, which can significantly improve gastric retention, reduce gastric residual volume, and improve gastric emptying function, so as to alleviate GI motility disorders (23–25). According to the results of this study, we hypothesize that the protective effects of long-term tuina on GI function in DM and inhibition of DGP might be associated with upregulating Piezo2 expression in intestinal epithelial EE cells and promoting 5-HT release from EC cells. However, further mechanistic studies with Piezo2/5-HT pathway inhibitors are required to confirm this causal relationship. Unfortunately, we did not adequately address alternative mechanisms or establish causation. Our findings only demonstrate that long-term tuina preserves GI function in DM and inhibits DGP development, which is associated with the upregulation of Piezo2/5-HT pathway-related proteins in the ileum.

In addition, during tuina therapy, mechanical stimulation may regulate nerve conduction and autonomic nervous system balance by activating Piezo1 ion channels or Transient Receptor Potential Vanilloid (TRPV) ion channel family members. A study indicates that the Piezo1 mechanosensor is specifically expressed in cholinergic enteric neurons and is directly involved in the regulation of GI motility (57). Optogenetic stimulation of Piezo1^+^ cholinergic enteric neurons drives colonic motility, while Piezo1 deficiency reduces cholinergic neuronal activity and slows peristalsis. Additionally, Piezo1 deficiency in cholinergic enteric neurons abolishes exercise-induced acceleration of GI motility (57). Another study showed that TRPV4 is expressed and functional in intestinal epithelial cells; its activation in the GI tract causes increases in intracellular calcium concentrations, chemokine release, and colitis (58).

The molecular mechanisms, signaling pathways, and downstream effects by which tuina activates channels such as Piezo and TRPV remain unclear, and further in-depth investigation will require the development of additional animal models and cell-based experiments. Currently, clinical trials of tuina for DGP have relatively small sample sizes. Future multi-center, large-scale randomized controlled trials are needed to validate the long-term efficacy and safety of tuina. Therefore, future research should focus on the molecular mechanisms and signaling cascades of tuina activating mechanically sensitive ion channels, as well as multi-center, large-sample randomized controlled clinical trials. Simultaneously, it should explore the synergistic effects of tuina with other therapeutic interventions and the development of individualized treatment plans, providing theoretical support and practical evidence for the precision treatment of DGP.

This study has some limitations. First of all, we used authentic hands-on tuina techniques. The operators were trained uniformly, and the procedures, manipulation frequency, and duration of tuina manipulations were restricted. We conduct tuina therapy with a level of force that ensures the comfort of the rats. This is similar to the actual effort in clinical settings. The downside is that it is impossible to accurately quantify the force of the tuina. Secondly, this study has a sample size of five rats per group, which may pose a risk of result bias due to the relatively small sample size. Thirdly, owing to certain reasons, this study did not set up control groups with Piezo2 inhibitors, 5-HT antagonists, or Piezo2 knockout mice. The mechanism by which long-term tuina upregulates the mechanoreceptor Piezo2 and promotes the release of 5-HT from EC cells has not been elucidated. In future research, we will further refine the experimental design and conduct in-depth mechanism studies.

## Conclusion

In conclusion, our study suggests that the protective effects of long-term tuina on GI motility are associated with the modulation of CaM, MLCK, nNOS, Piezo2, 5-HT, and 5-HT4R. A potential mechanism may involve Piezo2-mediated mechanical sensitivity and 5-HT signaling, but causal validation is required. This study provides not only evidence for tuina therapy as a potential alternative therapy for the prevention and treatment of DGP but also a basis for further research on the potential mechanism of tuina therapy in the prevention and treatment of DGP. Future studies should explore how tuina therapy can play a protective role in the GI tract and inhibit the occurrence and development of DGP through the mechano-chemical signal coupling of the Piezo2/5-HT pathway.

## Data Availability

The raw data supporting the conclusions of this article will be made available by the authors, without undue reservation.
