# Supplementary material for: Long-term tuina can inhibit the occurrence of gastroparesis by protecting gastrointestinal function in diabetic rats
Source: Front Endocrinol (Lausanne). 2025 Jun 25;16:1536567. doi: 10.3389/fendo.2025.1536567 (PMC12238824; doi:10.3389/fendo.2025.1536567)
Supplement: Supplementary file 1 [file DataSheet1.docx]

**Links to the raw data attachment database：**<https://www.jianguoyun.com/p/DaFlAUMQuqKIDRjM_t8FIAA>
